# Integration of physiology in a curriculum on human structure: a snapshot of the cardiovascular block

**DOI:** 10.3389/fphys.2023.1236409

**Published:** 2023-07-14

**Authors:** Rosemary B. Bassey, Robert V. Hill, William P. Rennie

**Affiliations:** Department of Science Education, Donald and Barbara Zucker School of Medicine at Hofstra/Northwell, Hempstead, NY, United States

**Keywords:** integrated curriculum, physiology, assessment, spiral integration, undergraduate medical education

## Abstract

With the gradual shift from discipline-based to competency-based medical education, the integrated curriculum has become a popular model for connecting basic science and clinical content in undergraduate medical education. Despite its popularity, there are concerns that important physiological concepts are not adequately addressed. We describe the spiral integration of physiology content in the 5-week Cardiovascular block of our Homeostasis course at the Zucker School of Medicine. We also describe our approach to incorporating physiology into an integrated, constructed response, short-answer assessment format. Our approach to spiral integration consists of rotating lab stations that highlight the distinction between normal and abnormal states, linked with appropriate clinical interventions. Physiology is at the core of integration in any curriculum and the basis of all applied fields of medicine, hence our approach is that teaching structural relationships would not be valuable without consideration of its functions, which can then be utilized in discussion of clinical presentations, imaging, and relevant pathologies. Likewise, our integrated assessments require the students to compose their answers to the questions from scratch, which creates a shift in mode of students’ preparation from rote memorizations to more cognitive processing that enhances critical thinking.

## 1 Introduction

Due to rapid technological advancements and ever-changing healthcare needs, there has been a collective call for training future physicians to be more coordinated and prepared for a dynamic healthcare setting (Frenk et al., 2010; Wartman and Combs, 2018; Shrivastava and PS, 2020). As a result, undergraduate medical curricula have gone through a series of evolutions in the last century. First, the Carnegie Foundation for the Advancement of Teaching, influenced by Abraham Flexner’s 1910 vision, spearheaded the move for major reforms in medical education which gave rise to the 2+2 curriculum (Irby et al., 2010). The 21st century began with another call by the Carnegie Foundation for reforms in medical education due to the failure of the 2+2 curriculum to integrate both basic and clinical sciences (Drake, 2014). This was followed closely by the American Medical Association’s “accelerating change in medical education” initiative which provided infrastructure and resources to support innovations in medical education (McBride and Drake, 2018). Since then, there have been several recommendations designed to integrate basic sciences at various levels of undergraduate medical education (UME) (Wijnen-Meijer et al., 2020).

The integrated curriculum has become an increasingly popular model in medical education over the years, signifying the shift from content-based to a more outcome- and competency-based education (Wijnen-Meijer et al., 2020; Hense et al., 2021). Shoemaker defined it as “education that is organized in such a way that it cuts across subject matter lines, bringing together various aspects of the curriculum into meaningful association to focus upon broad areas of study.” (Shoemaker, 1989) Based on various approaches to implementation, integrated curricula are classified as having horizontal, vertical, and spiral integration (Brauer and Ferguson, 2015; Haudek et al., 2022). In horizontal integration, all the basic science disciplines are taught concurrently within a predetermined period whereas in vertical integration, both basic and clinical sciences are combined across time through a stepwise increase of knowledge alongside graduated clinical responsibilities (Brauer and Ferguson, 2015; Wijnen-Meijer et al., 2020; Amini et al., 2021). Spiral integration combines elements of horizontal and vertical integration (Haudek et al., 2022).

The aim of curricular reforms in favor of integration of basic and clinical curricula is therefore to employ contextualized application-based learning which gives relevance to basic science and has been reported to improve motivation, enhance long-term retention, and develop skills useful for life-long learning (Ambrose et al., 2010; Brauer and Ferguson, 2015). Furthermore, assessments have a powerful influence on student learning, hence the successful implementation of an integrated curriculum comes with adopting the same deliberate planning and scrutiny of the design of integrated assessments (Fielding and Regehr, 2017). This has been shown to take different forms including clinical reasoning and case-based problems (Brauer and Ferguson, 2015).

Physiology is an important core component of the undergraduate medical curriculum (Sefton, 2005), and sufficient knowledge of physiology is required to develop competent physicians (Abdul-Ghaffar et al., 1999; Hasan and Sequeira, 2012). The Zucker School of Medicine, founded in 2008, established a framework for spiral integration at the program, course, and session levels, along with a complementary assessment system, strategically designed with the collaboration of both basic science and clinical faculty (Ginzburg et al., 2015). Because the design included no discrete course on physiology, this important basic science content was deliberately integrated at multiple touchpoints throughout the curriculum. Here, we describe one example of the integration of physiology content in the pre-clerkship curriculum at the Zucker School of Medicine.

## 2 Method

The pre-clerkship curriculum at the Zucker School of Medicine, termed the “First 100 Weeks” (FOW), includes seven courses designed around specific body systems and concepts related to their function in health and disease. Each course includes three curricular components. Mechanisms of Health, Disease, and Intervention (MHDI) covers foundational basic science areas such as biochemistry and cell biology, and the scientific basis for pharmacologic treatments. Patient, Physician, and Society (PPS) addresses clinical skills, communications, and related bio-psychosocial aspects of the medical profession. The third curricular component, Structure, encompasses gross anatomy and related disciplines, including embryology, histology, pathology, and medical imaging. Once a week, we offer laboratory sessions designed to reinforce and complement the material in the other two components. Within a given course, these components are all vertically integrated such that material presented at each session complements other sessions throughout the day, week, and course.

The functional units of our Structure curriculum are 30-min stations, specifically designed to integrate with one another over the course of a 2-h lab session. Half the class (*n* = 50) typically rotate through four stations, such that there are 12 or 13 students at each station at a given time. Stations are usually team-taught with at least two facilitators, for an effective student-to-faculty ratio of 7:1. Depending on each station’s primary focus, facilitators may be basic science faculty (anatomists) or practicing clinicians in various disciplines (e.g., surgeons, radiologists, pathologists).

Attendance is expected at all lab sessions, and students are expected to prepare by reviewing a lab guide with learning objectives and suggested readings or audiovisual materials. Stations are framed with higher order questions to elicit discussion about the learning objects. Socratic questioning is employed to probe the depth of student understanding, and expose (and fill) knowledge gaps (Elkowitz, 2021). Slide decks released after the session contain suggested answers to adequately address the learning objectives, along with links to additional resources.

While half the class rotates through lab stations, the other half participates in an “Alternate Time” session. The alternate time (or “Alt. Time”) sessions take a variety of forms, including complementary large group sessions, ultrasound practice, clinical skills, or dissection. The two groups switch after a short break, so that all students experience both the lab stations and the alternate time session. Student groups are semi-randomly assigned, and students remain with their Structure lab group for the entire year, mainly to promote team building around dissection experiences.

Based on student evaluations and faculty observations, one area in which physiology is particularly well-integrated is our Homeostasis (HOM) course. This final course of the first-year curriculum covers the cardiovascular, pulmonary, and renal systems, and highlights the interplay between these closely related systems. Although multiple systems are considered at each session, the HOM course can be construed as having a 5-week block on cardiovascular topics, followed by two 2-week blocks on the remaining systems. The 5-week cardiovascular block includes five Structure sessions, summarized in Table 1. Below we present a detailed description of each week’s content, contextualize the Structure component, and highlight instances where physiology content has been deliberately integrated into learning activities. These descriptions therefore approach integration at the week, session, and station levels.

**TABLE 1:** Integration of physiology into Structure sessions during the 5-week cardiovascular block of the Homeostasis course.

Week	Session title	Major area of physiology integration	Instructional resources and methods
1	Reintroduction to cardiac structure	Preload, afterload, LVEDP/V, stroke volume, ejection fraction, cardiac output, Frank-Starling Curve; concentric and eccentric hypertrophy	Prosected donor hearts, normal compared with those demonstrating valvular pathology and/or adaptive changes in wall thickness and chamber size
2	Pericardial and mediastinal structure	Clinical reasoning station: Fluid overload, constrictive pericarditis, comparison with tamponade, and phenomena such as ventricular interdependence, equalization of diastolic pressures, and Kussmaul’s sign	Socratic dialogue; progressive disclosure of examination findings, laboratory data, imaging, and cardiac catheterization
3	Normal and abnormal vascular structure	Circulatory adaptations at birth and congenital defects of the heart and great vessels: physiologic role of fetal shunts, physiologic consequences of cardiac defects, physiologic basis of shunt reversal, and physiologic implications of ultrasound waveforms in health and disease	Fetal and adult heart specimens; Real-time peripheral vascular ultrasound with spectral doppler tracings
4	Coronary circulation in health and disease	Structure of MI: physiologic features of EKG; echocardiography for assessment of chamber size, contractility, and flow characteristics	Right-sized hearts with wraparound EKG tracings; Real-time echocardiography in multiple planes
5	Flow through the heart and valvulopathies	Clinical reasoning large group session: Libman-Sacks endocarditis—heart murmurs, valvular calicific changes, Doppler flow indicating stenotic or regurgitant physiology, and Wiggers diagrams	Socratic dialogue; progressive disclosure of examination, laboratory findings, and imaging. Pathologic heart sounds, phonocardiograms, echocardiograms

### 2.1 Week 1: heart, lungs, and kidneys—a dynamic trio

This curricular week introduces the three systems (albeit with a focus on the cardiovascular system) through small group, case-based learning, and large group sessions.

In the Structure lab, students study the detailed structure of the heart from multiple different points of view. Since students have already studied cardiovascular structure superficially in their introductory course, this session is titled “Reintroduction to Cardiac Structure.” The lab session includes four stations: (i) Cardiac Form and Function, (ii) Cardiac Histology, (iii) Approach to the Chest X-ray, and (iv) Cardiac Anatomy and Relationships. Half the class (*n* = 50) rotates through these stations, spending approximately 30 min at each station. The other half of the class begins with an “alternate time” large group session on cardiac embryology. The two groups switch after a short break, so that all students experience both the large group session and the four lab stations.

Physiology is most prominently integrated into the station “Cardiac Form and Function.” At this station, students are called upon to explain the concepts of preload, afterload, LVEDP/V, stroke volume, ejection fraction, and cardiac output. Using prosected heart specimens from donor bodies, they relate the effects of altered preload and afterload to heart morphology. With this foundation, they then systematically evaluate heart specimens for general morphology, signs of chamber enlargement, myocardial wall thickening or damage/defects, valvular stenosis or widening, and aortic dilation, and relate these to associated clinical presentations. Finally, they explain the physiologic changes underlying the Frank-Starling ventricular function curve and the effects of preload, afterload and inotropy on the family of curves representing different degrees of heart failure.

### 2.2 Week 2: electrical stability and instability: cardiac wiring

In the second week students explore the electrical system of the heart from the initiation of the action potential through the appearance of electrical activity as seen on an electrocardiogram. Small group case studies focus on arrhythmias, complemented by large group sessions on the pharmacology of antiarrhythmics.

This Structure lab again comprises four stations: (i) Pericardial Pathology, (ii) Clinical Reasoning, (iii) Anatomy of the Mediastinum, and (iv) Cardiac and Thoracic Imaging. During the alternate time, teams of students perform an exploratory dissection of the thoracic wall and cavity of their assigned donor body.

Physiology is deliberately integrated into the Clinical Reasoning station, where students reason through a case of constrictive pericarditis adapted from the New England Journal of Medicine Clinical Problem-Solving series (Reed et al., 2011). A faculty facilitator frames the case as largely a hypothesis-driven approach to the analysis of a physiologic problem: fluid overload. Following the published case’s structure, the facilitator then discloses details to compel the students to develop cardiac, hepatic and renal hypotheses. Physical findings such as increased JVP are related to the physiology of both cardiac tamponade and constrictive pericarditis and phenomena such as ventricular interdependence, equalization of diastolic pressures, and Kussmaul’s sign are explained. Through Socratic dialogue, the students eventually arrive at the correct anatomical diagnosis and (more importantly) an understanding of its physiologic basis.

### 2.3 Week 3: circulation and its control

The third week highlights the features of normal and abnormal circulation. Large group sessions on congenital heart defects and peripheral artery disease complement small-group cases on vascular pathologies.

Students rotate through four stations in this week’s Structure session: (i) Circulatory Adaptations at Birth, (ii) Normal Vascular Histology and Atherosclerosis, (iii) Congenital Defects of the Heart and Great Vessels, and (iv) Clinical Aspects of Aneurysm and Dissection. The alternate time comprises two additional activities: physical diagnosis and ultrasound of the peripheral vasculature.

This Structure session features numerous instances where physiology content has been integrated. For example, at the station “Circulatory Adaptations at Birth,” learners are asked to draw the fetal circulation—beginning, and ending at the placenta, and to rank fetal circulatory structures in order of oxygen content in the blood. This exercise highlights the physiologic role of fetal shunts such as the ductus arteriosus in ensuring the most oxygenated blood is preferentially delivered to the developing brain. Similarly, the station “Congenital Defects of the Heart and Great Vessels” emphasizes the physiologic consequences of cardiac defects such as VSD, transposition of the great vessels, and Tetralogy of Fallot and the physiologic basis of shunt reversal and Eisenmenger’s syndrome. In the alternate time session, students use ultrasound to acquire images of their classmates’ peripheral vascular flow in real time. The physiologic implications of ultrasound waveforms in health and disease are discussed, and peak systolic and diastolic flow are measured via spectral doppler applications.

### 2.4 Week 4: ischemia

The fourth week of the Homeostasis course focuses on ischemia in the heart, lungs, and periphery. Small-group case-based discussions highlight hypercoagulation as well as the generation and consequences of atherosclerosis. Large group sessions focus on microcirculation and pharmacological regulation of vascular tone, cardiovascular diseases, and the management of acute and chronic ischemia.

Structure lab this week entails three stations: (i) Coronary Circulation, (ii) The Structure of Myocardial Infarction, and (iii) Pathology of Ischemic Heart Disease. In the alternate time, students participate in a session on echocardiography, acquiring and measuring images of their classmates’ hearts.

Physiology is integrated throughout this week’s lab stations, perhaps most notably in the station “Structure of Myocardial Infarction.” At this station, students directly relate anatomical structures such as coronary arteries and the conductive system to indicators of altered physiology on EKGs. In an innovative exercise, students wrap paper EKG strips around human donor hearts of just the right size to demonstrate the relationship between physiologic features of EKG and the underlying anatomical structures responsible. Ischemic effects on SA and AV nodal tissue and bundle branch blocks as seen on characteristic EKG waveforms are related to the vascular supply of these areas in both right and left dominant circulations. In the alternate time, students perform echocardiography in multiple planes for assessment of chamber size, and contractility and use color Doppler imaging to demonstrate flow through the aortic and mitral valves, measure the aortic root, and compare their findings with pathologic images.

### 2.5 Week 5: normal and abnormal pumping: cardiopulmonary hemodynamics

The fifth week examines the importance of the heart as a pump. Small group cases highlight cardiac pathophysiology and its treatment, while large group sessions focus on hemodynamics, valvular function, and the pathophysiology and pharmacological management of heart failure.

This week lacks an in-lab session, instead approaching Structure with two large-group sessions. The first focuses on the gross and histologic structure of heart valves, in both normal and disease states. The second describes flow through the heart and how it is altered in the context of specific valvulopathies. In the alternate time, a physical diagnosis session allows students to revisit techniques of the cardiac exam.

In the session on flow and valvulopathies, a faculty member leads students through a clinical reasoning exercise based on a NEJM case on Libman-Sacks endocarditis presenting as mitral stenosis (Tarter et al., 2013). Through progressive disclosure of case details, students use both causal and probabilistic reasoning to apply the observed physiologic changes to the underlying disease process. Following the clinical reasoning exercise, students listen to and interpret specific heart murmurs, relating these to echocardiographic images depicting changes of chamber size, valvular calicific changes, and Doppler flow indicating either stenotic or regurgitant physiology, including such aspects as the venturi effect on the mitral valve with late-stage aortic insufficiency. Wiggers diagrams are used to trace the pressure/volume relationships and the generation of murmurs for various pathologies affecting primarily the aortic and mitral valves.

### 2.6 Integration of physiology in assessment

Curricular integration of basic and clinical sciences at the assessment level entails the design of appropriate tools that facilitate application of knowledge (Kulasegaram et al., 2013). It is therefore imperative to align our assessment strategies with the curricular approaches, and ensure every component is well represented (Gulikers et al., 2004). At the Zucker School of Medicine, we employ case-based, open-ended short essay questions in alignment with our self-directed integrated curriculum in a way that facilitates critical thinking and problem-solving skills. Many of our questions include medical imaging studies, histology slides, or gross pathology images and thus require an integrated approach to answering. An additional facet of our Structure laboratory exam is the inclusion of prosected donor bodies, isolated organs, or pathology specimens. These exams expand on the traditional “spotter” or “bellringer” anatomy lab exam, wherein students typically must identify structures by name (Brenner et al., 2015; Choudhury and Freemont, 2017) or answer second-order questions based on their recognition of a structure. Our integrated assessments require students to link multiple observations and make predictions based on the underlying physiology. The following is an example of an assessment question from our Structure cardiovascular block that integrates anatomy, physiology, and ultrasound imaging, couched in a clinical vignette and using an anatomic specimen (dissected donor heart with the aortic valve indicated with a white pin).

Phil is a 26-year-old man whose primary care doctor has been following a suspicious heart murmur for the last 3 years. An echocardiogram has now been performed and detects regurgitant flow through the valve indicated with the white pin in the donor specimen.

· Predict which chamber of the heart would be affected by regurgitant flow through this valve.
· Describe two echocardiographic observations you would expect to find and explain their physiologic consequences.

A suggested full-credit answer to this question is as follows: This is the aortic valve. Regurgitant flow through this valve will primarily affect the left ventricle. In this young patient with a “suspicious” murmur, it is possible the doctor was considering Marfan’s syndrome, which is characterized by chronic aortic insufficiency. Echocardiographic findings in this patient would include a dilated left ventricular chamber, likely with normal wall thickness, representing eccentric hypertrophy. The outflow tract would show active flow in both systole and diastole due to failure of the aortic valve to close.

The subsequent regurgitation will be visible using the color Doppler flow application. The aortic root may be seen as dilated, preventing coaptation of the valve cusps and resulting in regurgitant flow. Mitral valve dysfunction (involving the anterior leaflet) may also be seen as a result of the Venturi effect of the regurgitant flow through the aortic valve during diastole. In late stages the left ventricular dilation may involve the mitral annulus, promoting regurgitant flow through this valve and subsequent left atrial enlargement, both possible findings on echo. The physiologic response to aortic regurgitation and its associated increased preload is eccentric hypertrophy of the left ventricle in an attempt to increase contractility through the Frank-Starling mechanism. Over time this will be unable to compensate, leading to systolic heart failure characterized by a wide pulse pressure and its associated clinical findings. Further effects on the mitral valve as noted above can lead to volume overload on the left atrium, increased pulmonary hydrostatic pressure and subsequent pulmonary edema.

The question is then scored anonymously according to an analytic rubric. This approach allows for partial credit to be awarded, even if the initial identification is incorrect. The rubric for this question is worth three points, awarded as follows: 1 point: answer states that left ventricle would be the chamber affected. 1 point: Answer cites TWO predicted echocardiographic findings, ideally 1) bidirectional flow through the aortic valve noted with color doppler and 2) dilated left ventricle. (Credit also awarded for dilated left atrium IF explained as per suggested answer). 1 point: Answer cites a physiologic response of ECCENTRIC hypertrophy (not just “left ventricular hypertrophy”) and any appropriate physical finding of aortic insufficiency, or description of end-stage mitral, left atrial, or pulmonary consequences. (NOTE: If answer misidentifies aortic valve as pulmonic valve, however ALL echo and physiologic consequences are consistent with regurgitation through pulmonic valve, then 1 point maximum is awarded).

This is just one example of an open-ended, constructed response (i.e., “essay”) question from one of our assessments. While this particular question focuses on anatomy and physiology, content from other disciplines are frequently integrated. A single question may therefore test learning objectives from multiple stations, sessions, or weeks. Structure assessments typically comprise around 30–35 such questions administered in two parts on the same day. Grades earned are either “meets expectations” (ME; equivalent to a passing grade), “meets expectations with recommendations” (MWR; akin to a marginal pass), or “does not meet expectations” (DNM; a failing grade). Students self-assess in a secure exam review period by comparing their answer with suggested responses. Those with a grade of DNM receive support from course directors and other support personnel. Faculty meet with any students who seek clarification on concepts and the awarding of partial credit under analytic grading rubrics.

## 3 Discussion

Physiology is a fundamental discipline in medical education which needs to be integrated with other medically relevant content essential for clinical practice (LeClair et al., 2023). In this paper, we described the various forms of physiology integration in the cardiovascular block of our Homeostasis course. As described earlier, the Structure component is the amalgamation of various disciplines including anatomy, embryology, histology, pathology, and medical imaging. Our approach to spiral integration includes stations highlighting the distinction between normal and abnormal states, linked with appropriate clinical interventions. The determination and organization of these fully integrated stations are based on their interrelatedness and interdependencies to the subject discussed. The selection of content in an outcome- and competency-based program must also consider the required competencies to be developed by the learners at the end of their training. Physiology is at the core of integration in competency-based curricula and forms the basis of all applied fields of medicine (Nathial, 2020). Moreover, many common diagnostic tests are based on measurement of key physiological indicators which can give insight into the pathophysiology of disease (Joyner, 2011).

Accordingly, in the first week of our cardiovascular block, cardiac macro- and micro-structure, development, and structural relationships are all purposefully considered in the context of physiologic function, which can then be utilized in discussion of clinical presentations, various thoracic imaging modalities, and relevant pathologies. The subsequent weeks follow a similar trajectory, albeit with some variations in session design depending on the content covered. Our design of the Homeostasis course is consistent with the spiral integrated curriculum model which integrates normal structure and function, then progresses to abnormal structure, function, clinical interventions, alongside experiential training (Harden, 1999). For instance, the first week began with a session on cardiac embryology along with associated congenital defects which was revisited in the third week in the “congenital defects of the heart and great vessels” station along with consideration of physiological consequences of these defects. The spiral approach to our curriculum design ensures that the students acquire knowledge through reinforcement of previous material and graduated complexities of the content with each session (Baynouna Al Ketbi, 2018). This recurring, yet progressive approach to integrated curriculum design is believed to promote effective knowledge acquisition and retention (Brauer and Ferguson, 2015). It is also important to note that this curriculum is not static and thus frequent reconsideration and adjustments of content, and active methods of content delivery are required to facilitate an upward continuum of learning (Harden, 1999).

The successful incorporation of physiology in the integrated curriculum depends on the adoption of integrated assessment styles. It is an established notion that assessment drives learning (Norcini et al., 2011), with consideration of assessment for learning gaining more prominence due to the role feedback plays in transforming formative assessments into learning (Konopasek et al., 2016). Additionally, more attention should be given to designing case-based questions which require more cognitive processing and problem-solving skills (Schuwirth and Van der Vleuten, 2011). At the Zucker School of Medicine, we employ an integrated, open-ended response essay exam format in both our formative and summative assessments during the pre-clerkship years which aligns with the criteria in assessment for learning (Norcini et al., 2011). These constructed response short-answer questions require the students to compose their answers to the questions from scratch, which creates a shift in mode of students’ preparation from rote memorizations to the use of deep learning and critical thinking strategies (Cilliers et al., 2012; Hift, 2014; Olvet et al., 2022). Likewise, it models the fundamentals of the integrated curriculum by entailing students to apply basic science knowledge to clinical scenarios (Bird et al., 2019). As with the curriculum itself, our assessments are reviewed as a team to ensure clarity and that they accurately reflect the learning objectives. The questions and accompanying rubrics also undergo frequent reviews based on students’ responses and performance (Olvet et al., 2022).

## 4 Conclusion

Physiology is a fundamental discipline in medicine and should be at the core of integrated curriculum planning to ensure meaningful incorporation of basic and clinical science content. This requires the involvement of both basic and clinical sciences faculty to ensure relevance, interrelatedness and prevent content overload. Likewise, careful design of appropriate assessment tools is critical for successful implementation of these curricular changes, as cognitive processing and effective contextualization must complement factual knowledge for it to be successfully applied. Presented here is just one example of deliberate integration of physiology in our program. Numerous additional instances exist in our First 100 Weeks pre-clerkship curriculum and in newly developed sessions during the core clerkships.

## Data Availability

The original contributions presented in the study are included in the article/Supplementary Material, further inquiries can be directed to the corresponding author.
